# Drug-induced premature senescence model in human dental follicle stem cells

**DOI:** 10.18632/oncotarget.14085

**Published:** 2016-12-21

**Authors:** Yuanfen Zhai, Rongbin Wei, Junjun Liu, Huihui Wang, Wenping Cai, Mengmeng Zhao, Yongguang Hu, Shuwei Wang, Tianshu Yang, Xiaodong Liu, Jianhua Yang, Shangfeng Liu

**Affiliations:** ^1^ Department of Ophthalmology, Shanghai Tenth Peoples Hospital, Tongji University School of Medicine, Shanghai, P. R. China; ^2^ Department of Pediatric Dentistry, School of Stomatology, Tongji University, Shanghai Engineering Research Center, Shanghai, P. R. China; ^3^ Department of Stomatology, Huashan Hospital, Fudan University, Shanghai, P. R. China; ^4^ Department of Neurosurgery, Huashan Hospital, Fudan University, Shanghai, P. R. China

**Keywords:** aging, dental stem cells, cellular senescence model, stress, DNA damage, Gerotarget

## Abstract

Aging is identified by a progressive decline of physiological integrity leading to age-related degenerative diseases, but its causes is unclear. Human dental pulp stem cells (hDPSCs) has a remarkable rejuvenated capacity that relies on its resident stem cells. However, because of the lack of proper senescence models, exploration of the underlying molecular mechanisms has been hindered. Here, we established a cellular model utilizing a hydroxyurea (HU) treatment protocol and effectively induced Human dental pulp stem cells to undergo cellular senescence. Age-related phenotypic changes were identified by augmented senescence-associated-β-galactosidase (SA-β-gal) staining, declined proliferation and differentiation capacity, elevated G0/G1 cell cycle arrest, increased apoptosis and reactive oxygen species levels. Furthermore, we tested the expression of key genes in various DNA repair pathways including nonhomologous end-joining (NHEJ) and homologous recombination (HR) pathways. In addition, our results showed that Dental pulp stem cells from young donors are more resistant to apoptosis and exhibit increased non-homologous end joining activity compared to old donors. Further transcriptome analysis demonstrate that multiple pathways are involved in the HU-induced Dental pulp stem cells ageing, including genes associated with DNA damage and repair, mitochondrial dysfunction and increased reactive oxygen species levels. Taken together, the cellular model have important implications for understanding the molecular exploration of Dental pulp stem cells senescence and aging.

## INTRODUCTION

Aging generally experience a gradual loss in physiological integrity and rejuvenated capacities within adult tissues [1], which lead to impaired function, increased susceptibility to disease and elevated risk of death [2]. Hence, homeostasis of tissues is lost since cells enter senescence by one of the mechanisms they indicated. The rate of aging is regulated evolutionarily conserved signaling pathways [3, 4]. The general mechanism of aging is widely considered as the chronic accumulation of cellular damage [4, 5]. The ability of adult stem cells to transition from a inactive quiescent state (G0) to an active proliferative state (G1, S, G2 and M) is paramount to replenish the tissue, and an imbalance in these two states may lead to pathologic consequences [6]. The mechanisms for aging-relative stem cell decline is completely unclear. Damaged DNA accumulation in aged stem cells may constantly arise from internal and external factors including DNA replication, transcription, recombination errors, repair, spontaneous chemical reactions and assaults metabolism-derived agents [7]. Rube C. found that aged human hematopoietic stem cells (HSC) have compromised capacity to repair experimentally induced DNA damage, such as ionizing radiation and other clastogens [8]. In addition, the mechanistic basis by which increased levels of DNA damage in aged stem cell can decrease stem cell dysfunction are still being illustrated. Genetic assaults may increase stem cell ageing or apoptosis and may directly trigger gene regulation, causing alterations in stem cell differentiation and self-renewal.

Recent studies on the causes (or stresses), signaling networks and machinery underlying the different types of cell ageing is still initial stage and current insights are largely using cell culture experiments. Further, damaged DNA accumulation and DNA insufficient repair often accompanied by stresses (oxidative stress and mitochondrial dysfunction) stimulate stem cell senescence and aging [9]. Oppositely, genomic integrity which is maintained by DNA repair pathways may defer cell senescence and aging [10]. Studies have shown that mutations in DNA repair genes might result in human monogenic progeroid syndromes (PS), and its deficiency may also lead to premature aging in mouse models [11]. Resident stem cell senescence and eventual exhaustion were triggered by DNA damage accumulation. Genotoxic insult with DNA repair pathway components significantly influence resident stem cell integrity throughout principal machinery in murine model [11]. For instance, aged hematopoietic stem cells (HSCs) and muscle stem cells show a higher levels of nuclear foci which was stained by the phosphorylated form of H2AX (γH2A.X) [8, 12, 13], which is a highly sensitive technique to detect DNA double-strand breaks, and this has shown that the kinetics of γH2AX-foci loss strongly correlate with the time course of DNA double-strand breaks (DSB) repair [14] . DNA damage accumulation in these stem cells may also be characterized through single cell gel electrophoresis assays [13, 15]. Further, hematopoietic stem cells (HSCs) deficiency and hematopoietic abnormalities in aged hematopoietic stem cells may result from DNA damage accumulation and defects in DNA damage repair pathway[12, 16]. In addition, DNA assaults in stem cells may reproduce both daughter stem cells and downstream lineages. Thus, DNA damage in stem cells can compromise cellular function such as genomic instability and their offspring.

In addition, the age-associated increase in reactive oxygen species has been considered as a causal factor of the ageing progression [17], while mitochondrial dysfunction is viewed an indicator of senescence [2], as a consequence of reactive oxygen species accumulation. This can take place through a lot of machinery; for instance, mitochondrial deficiency might trigger apoptotic pathway through elevating the propensity of mitochondria to permeabilize in response to stress [18] and drive inflammatory reactions by favoring reactive oxygen species-mediated and/or permeabilization-facilitated activation of inflammasomes [19].

In eukaryotes, the DNA repair mechanism has demonstrated to compromises damaged DNA, and it majorly contains a series of biologic processes including homologous recombination repair (HRR), nonhomologous end joining (NHEJ) (C-NHEJ and alt-NHEJ), mismatch repair (MMR), nucleotide excision repair (NER) and base excision repair (BER) [20]. The mismatch repair signaling pathway copes with DNA replication errors, containing mismatch base-pairing as well as nucleotide insertions and deletions. Double-strand DNA breaks are traditionally repaired by homologous recombination repair or non-homologous end joining, whereas single-strand breaks are mainly repaired by nucleotide excision repair or base excision repair [21]. The efficiency and activity of the DNA repair pathway seems to decline with advancing age, causing DNA damage accrual in different organs [20]. In mammals, genic mutations associated to the DNA repair mechanisms lead to phenotypic differences that share characteristics with age-related pathological conditions, containing metabolic and cardiovascular abnormalities, as well as being related to an elevated risk of malignancies and a shortened lifespan [20, 22]. Studies in many tissue systems suggest DNA repair can be in response to damaged DNA to avoid further genomic instability by cellular machinery [23]. Furthermore, The DNA damage response mainly was triggered by sensors (the MRE11-RAD50-NBS1 sensor complex, apical kinases ataxia-telangiectasia mutated (ATM), ataxia-telangiectasia- and RAD3-related (ATR) and DNA-dependent protein kinase), p53-binding protein 1), mediators (mediator of Claspin and breast cancer type 1, DNA damage checkpoint 1) and effectors (checkpoint kinase 1 (CHK1) and CHK2) [24]. These key proteins have been involved in either DNA repair and transient cell cycle arrest or damaged cellular elimination through apoptosis and/or senescence. Though stringent, the DNA repair pathways is not perfect and might result in misrepaired lesion accrual or genomic integrity [25].

In adult stem cells, DNA damage accumulation may result in the outcome of defective offspring, stem cell ageing or neoplastic transformation [26, 27], causing age-associated lack of tissue function and homeostasis [1]. It is now clear that activing DNA repair machinery in stem cells may delay the age-associated defective accumulation and then drive aged tissues to recover healthy function, particularly if such an intervention might happen before the establishment of damaged DNA in the genome [28]. In addition, Additional DNA damage repair/response systems under analogous inducible settings will be informative to access the full rejuvenation potential of these systems. These stresses share the similar situation in telomere damage is that they activate the DNA damage response, a signaling pathway in which apical kinases ataxia-telangiectasia mutated or ataxia-telangiectasia- and RAD3-related kinases block cell cycle process by stabilization of tumor protein p53 and transcriptional activation of p21. Similarly, activation of oncogenes serve also as a major cause of cell ageing. For instance, oncogenic Ras serves by overexpression of Cell Division Cycle 6 and suppression of nucleotide metabolism, leading to double stranded DNA breaks, aberrant DNA replication, and activation of the DNA damage repair signaling pathway [29, 30]. However, studies have shown that senescence result from activation of E2F3 or inhibition of c-Myc is DDR-independent involving p^19Arf^ and p16Ink4a [31, 32]. BRAF (V600E), also DDR-independent protein, triggers cell ageing by a metabolic machinery involving upregulation of mitochondrial pyruvate dehydrogenase [33]. Kondoh and Dörr et al. found that ageing is closely-related to profound metabolic balance [34, 35]. Moreover, several other experiments show that different tumour repressors drive a senescent growth arrest when inactivated, containing Neurofibromin 1, Phosphatase And Tensin Homolog, Retinoblastoma 1 and Von Hippel-Lindau Tumor Suppressor [31, 36]. Importantly, inactivation of Retinoblastoma 1(Rb) recruits the DNA damage repair [36], while the others are DDR-independent and serve by p19^Arf^ and p16^Ink4a^. A significant species-specific diversity is that ageing signaling pathways of mouse cells are much more dependent on p19^Arf^ than ageing in human cells [37]. Pedro Sousa-Victor et al. demonstrate that p16INK4a inhibition of Rb in geriatric stem cells triggers increasing ageing, based on the known capacity of the p16INK4a-Rb pathway to form ageing-relatived heterochromatic foci and accumulate DNA-repair foci staining by γH2AX, at the cost of the normalmyogenic fate [38]. In addition, Werner Syndrome RecQ Like Helicase (WRN) has a key part in recombination, transcription, repair, and DNA replication, as well as telomere maintenance, suggesting that the prominent inducer for Werner syndrome pathogenesis associated with genomic instability [2, 39]. Recent study show that WRN related to at least three proteins including HP1 Alpha Homolog (HP1a), Suppressor Of Variegation 3-9 Homolog 1(SUV39H1) and nuclear lamina-heterochromatin anchoring protein LAP2b. Targeted knock-in of catalytically inactive Suppressor Of Variegation 3-9 Homolog 1 in wild-type mouse stem cells recapitulates increasing cell ageing, resembling WRN-deficient mouse stem cells. Furthermore, decline in WRN and heterochromatin hallmarks are identified in mouse stem cells from advancing ageing individuals [40].

Biological understanding of ageing mechanisms mediating stem cell survival, self-renewal, self-repair, proliferation, differentiation and commitment to different cellular lineages is essential to defining the triggers and effectors of age-related stem cell disorganization. Moreover, those studies will help to determine interventions aimed at preventing and/or treating human diseases. Nevertheless, It is reasonable to propose that senescence accelerates the ratio of disease risk, resulting in the emerging insight of age-dependent disorders when mentioning conditions including metabolic, cardiovascular, and neurodegenerative diseases and tumorigenesis [41]. However, what triggers the senescence progression and how the different aspects of senescence biology affect health decline and disease incidence are initially to be understood. Because of the lack of proper model to demonstrate the important proteins and identify the molecular machinery with a relative short-period time. Dental stem cells can be obtained with ease, making them an attractive source for research. Therefore, it is vital to establish the vitro aging models using human DFSC to recapitulate DFSC ageing machinery. In addition, Hydroxyurea initially acted as an antineoplastic prescription [42] suppresse the ribonucleotide reductase and then decline the production of deoxyribonucleotides. Therefore, it could trigger DNA double-strand breaks near replication forks and act as a DNA replication inhibitor for mitochondrial and nuclear DNA, leading to cellular dysfunction and mitochondrial oxidative stress [43]. Here, we utilized hydroxyurea treatment to optimize and carefully characterize human DFSC ageing model that can recapitulate a number of the ageing attributes. Furthermore, RNA sequencing (RNA-seq) analysis unveiled molecular networks associated with different senescence markers, showing the broad utilities of our cellular model for mechanistic analysis of DFSC and human dental follicle ageing.

## RESULTS

### Persistent DNA damage induced by HU treatment

DPSCs from dental follicle of donors were cultured in DMEM/F12 medium with FBS and formed human dental follicle stem cells *in vitro*. Characterisation of DFSCs was performed using Human MSC Analysis Kit (BD) by flow cytometry (Unpublished data were provided by H.W. and J.L.).

**Figure 1:**
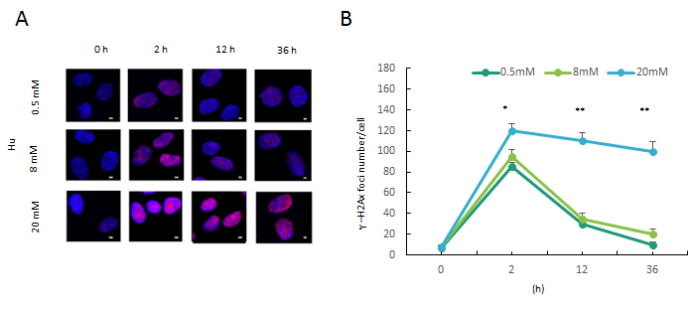
Persistent DNA damage induced by HU treatment **a**. Formation of DNA double-strand breaks in DFSCs post HU treatment. DFSCs were treated with 0.5, 8 and 20mM HU for for 2 or 12 h and and allowed to recover until 36 h post treatment. Cells were fixed and stained for γH2AX foci (red) at the indicated time points post HU treatment. Scale bar: 5 mm. **b**. Graphical depiction of the number of γH2AX foci in DFSCs treated with 0.5, 8 and 20mM HU over 36 h. Data are expressed as mean±S.E. from three independent experiments. *P < 0.05, **P < 0.01, two-way ANOVA with Fisher's post hoc test

In order to determine the right dose of hydroxyurea to trigger double stranded DNA breaks and cellular age, we treated DFSCs with three different doses of 0.5, 8 and 20mM for 2, 12 and 36 h. The sites of DNA damage was stained by γH2AX foci to quantify the extent of DNA double-strand breaks. DFSCs without hydroxyurea treatment showed a basic expression of γH2AX foci. DFSCs with 0.5mM hydroxyurea treatment for 2 or 12 h or with 8mM hydroxyurea treatment for 2 h triggered a transient higher level in γH2AX foci amount which decreased quickly and recovered the basic level after 36 h hydroxyurea treatment. DFSCs with 8 mM hydroxyurea treatment for 12 h drived rapid accumulation in γH2AX foci amount but that failed to descend even after 36 h hydroxyurea treatment (Figures 1a and 1b).

### Proliferation and differentiation of HU-treated DFSCs

The data showed that the low dose of hydroxyurea only induced transient double stranded DNA breaks, while the high dose of hydroxyurea was incompatible with DFSC survivals. Thus, we determine to treat DFSC with hydroxyurea (8mM hydroxyurea, 24 h) that established persistent double stranded DNA breaks (Figure 1a).

**Figure 2:**
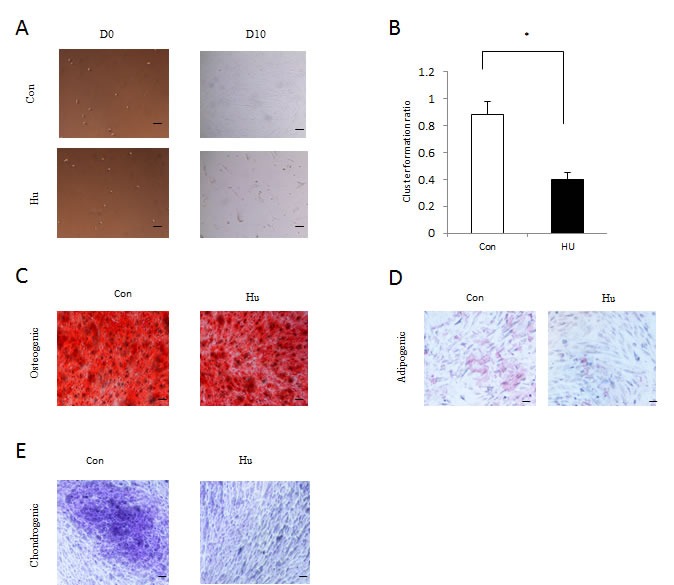
Proliferation and differentiation of HU-treated DFSCs **a**. *In vitro* proliferative capacity of DFSCs assayed by colony formation experiment. Representative images of colony from control (Con) and HU-treated (HU) cells. The number of colony formed after 10 days was counted. D0 shows the plated DFSCs after single-cell dissociation at day 0. D10 shows the colony formed after 10 days of culturing. Scale bar: 50 mm. **b**. *In vitro* proliferative capacity of DFSCs assayed by colony formation experiment. The number of DFSCs formed after 10 days was counted. Statistics for the ratio of DFSCs formation after 10 days *in vitro*. Mean±S.E. from three independent experiments. *P < 0.05, Student's t-test. **c**. After Osteogenic differentiation, DFSCs with and without HU treatment were analyzed by Alizarin Red staining after 3 weeks of culture (scale bar 50 μm). **d**. At day 14 after induction of differentiation, DFSCs with and without HU treatment was stained with Oil Red O (scale bar 50 μm). **e**. After Chondrogenic differentiation, DFSCs with and without HU treatment were stained with Alcian blue in combination with PAS (Periodic acid-Schiff) in an automated slide stainer and photo-documented (scale bar 50 μm).

To confirm whether a cellular DFSC aging model was establish with 8mM hydroxyurea treatment, we carried out a series of examinations. First, we performed clone formation assays and these data revealed that the ratio of dental follicle stem cell formation was dramatically declined through the hydroxyurea treatment (Figures 2a), revealing the self-renewal capacity of DFSCs is significantly declined with hydroxyurea treatment. Subsequently, we analyzed whether differentiation of DFSCs were affected with hydroxyurea treatment *in vitro*. The quantification of adipogenesis was identified by the percentage of cells that acquired fat droplets within two weeks. DFSCs with mild hydroxyurea treatment revealed consistently lower adipogenic differentiation than normal DFSCs (Figure 2b). Futhermore, we analyzed osteogenic differentiation by Alizarin Red staining of calcium phosphate precipitates, we also found the same result: DFSCs with mild hydroxyurea treatment revealed consistently lower osteogenic differentiation than normal DFSCs. In addition, chondrogenic differentiation showed the similar result (Figure 2). These results seem to support the notion, that DFSC with mild hydroxyurea treatment have a lower differentiation ability.

### Phenotypic characterization of mild HU-treated DFSCs

**Figure 3:**
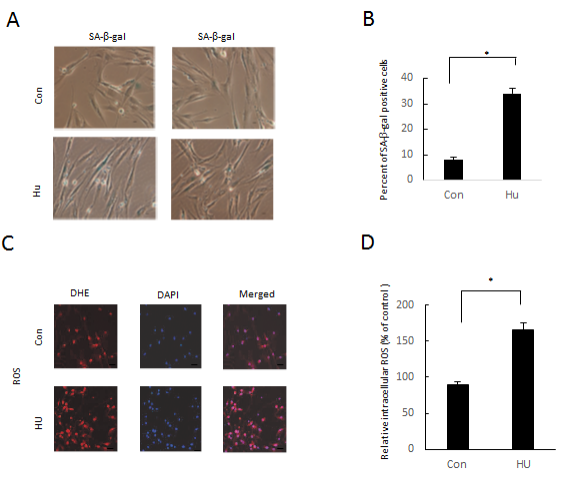
Phenotypic characterization of 8 mM HU-treated DFSCs **a**. Images of SA-β-gal^+^ DFSCs. with or without HU treatment. **b**. Statistics of the percentage of SA-β-gal^+^ DFSCs, mean±S.E. *P < 0.05, Student's t-test, Scale bar: 50 mm. **c**. ROS were detected using dihydroethidium (DHE) fluorescence. **d**. ROS level was normalized to that of control cells with statistics expressed as mean±S.E. *P < 0.05, Student's t-test. Scale bar: 60 mm.

To verify whether cell senescence was caused with the mild DNA damage treatment in human DFSCs, the human DFSCs were stained with senescence-associated-β-galactosidase. The percentage of positive senescence-associated-β-galactosidase DFSCs elevated from 8 % in the untreated cells to 34 % in the hydroxyurea treatment (Figure 3a). Cellular ageing is usually accompanied by elevated Reactive oxygen species level in cells. In accordance with that, DFSCs with hydroxyurea treatment showed significant increase of Reactive oxygen species level than the untreated cells (Figures 3c and 3d).

### Molecular characterization of mild HU-treated DFSCs

**Figure 4:**
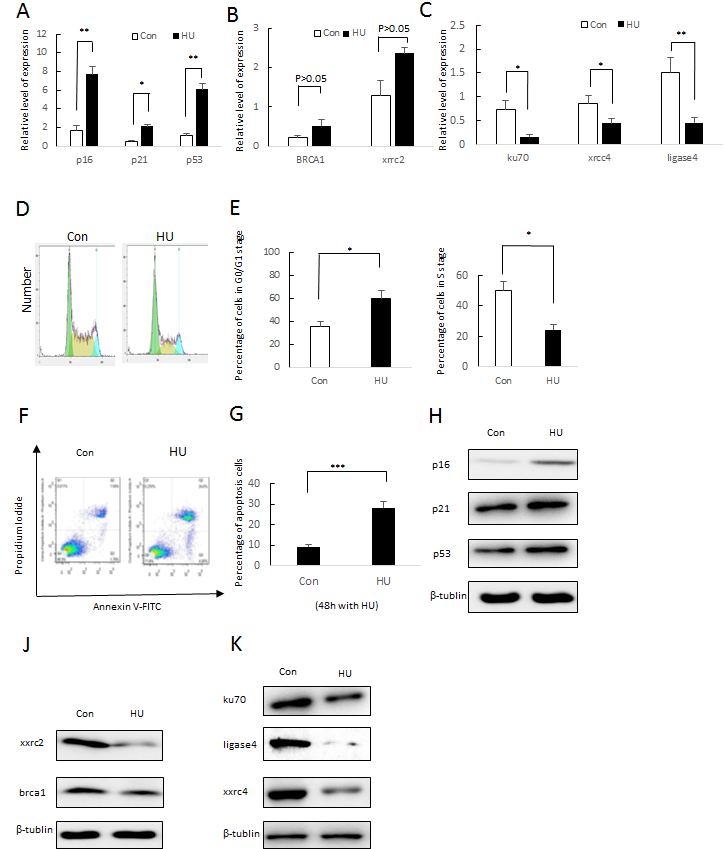
Molecular characterization of 8 mM HU-treated DFSCs **a**. qRT-PCR analysis of p16, p21 and p53 expression normalized by β-actin. Values represent mean±S.E. of two independent samples conducted in triplicate. *P < 0.05; **P < 0.01, Student's t-test. **b**. qRT-PCR analysis of HR (X-ray repair cross complementing 2 (xrcc2) and brca1) and NHEJ (ku70, ligase4 and X-ray repair cross complementing 4 (xrcc4)) gene expression normalized by β-actin **c**. Values represent mean±S.E. of two independent samples conducted in triplicate. *P < 0.05; **P < 0.01, Student's t-test. **d**. Cell cycle arrest of DFSC cells was were treated with 8 mM HU and without HU (Con). After 24 h of incubation, cells were harvested and stained with PI followed by Cellometer imaging and analysis. X-axis indicated the PI fluorescence intensity that correlates to the DNA content. **e**. FACS analysis and percentages of different cell stages are presented as mean of percentage±S.E. . **f**. DFSC cells were treated with HU (8 mM) and without HU for 48 h and examined by flow cytometry using Annexin-V/PI staining to label apoptotic cells. **g**. FACS analysis and percentages of apoptotic cells are presented as mean of percentage±S.E. .(f) Western blotting analysis of p16 and p21 expression. (g) Western blotting analysis of xxrc2 and brac1 expression. (j) Western blotting analysis of ku70, ligade4 and xxrc4 expression

Recent studies reported that p53/p21 and p16INK4A signaling pathway were involving in cellular response to damaged DNA. The two signaling pathways play a role in regulating cell cycle arrest and cellular ageing. As we expected, 8mM hydroxyurea inducement result in a higher expression level of p53, p21 and p16INK4A as observed by Quantitative Real-time Polymerase Chain Reaction and Western blotting (Figure 4a and 4h). In addition, some major genes that involving HR repair pathway (BRCA1 and xrcc2) non-homologous end joining repair pathway (X-Ray Repair Cross Complementing 6 (Ku70), Repair Cross Complementing 4 (xrcc4) and DNA ligase IV (LigIV)) were observed by quantitative real-time polymerase chain reaction

Furthermore, we present Fluorescence Activated Cell Sorting assays. As determined by propidium iodide (PI) staining, hydroxyurea inducement showed a notable increase in G0/G1 phases and a notable decline in S populations (Figures 4d and 4e). We used the Annexin-V FITC Apoptosis Detection Kit to analyze cell apoptosis. As is expected, 8mM hydroxyurea inducement increased apoptotic level in DFSCs compared to the control group (Figure 4f and 4g).

### Phenotypic and molecular characterization of mild HU-treated DFSCs between young and old

**Figure 5:**
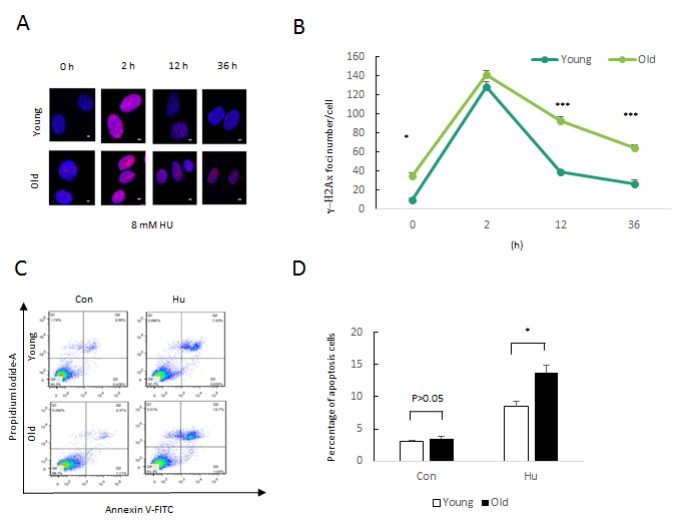
Phenotypic and molecular characterization of 8mM HU-treated DFSCs between Young and Old **a**. Formation of DNA double-strand breaks in DFSCs post HU treatment. DFSCs were treated with 0.5, 8 and 20mM HU for for 2 or 12 h and and allowed to recover until 36 h post treatment. Cells were fixed and stained for γH2AX foci (red) at the indicated time points post HU treatment. Scale bar: 5 mm. **b**. Graphical depiction of the number of γH2AX foci in DFSCs treated with 0.5, 8 and 20mM HU over 36 h. Data are expressed as mean±S.E. from three independent experiments. *P < 0.05, **P < 0.01, two-way ANOVA with Fisher's post hoc test. **c**. DFSC cells were treated with 8 mM HU for 48 h and examined by flow cytometry using Annexin-V/PI staining to label apoptotic cells. **d**. FACS analysis and percentages of apoptotic cells are presented as mean of percentage±S.E. .

To determine phenotypic characterization of mild HU-treated DFSCs between Young and Old, DFSCs from young donors and old donors were treated for 2, 12 and 36 h at a dosing of 8mM. The sites of DNA damage was stained by γH2AX foci to quantify the extent of DNA double-strand breaks. DFSCs from young donors and old donors showed a basic expression of γH2AX foci. DFSCs from young donors with 8mM HU inducement for 2 or 12 h or with 8mM HU inducement for 2 h induced a transient higher level in γH2AX foci amount that decreased rapidly and recovered the basic level after 36 h HU inducement. DFSCs from old donors with 8 mM hydroxyurea inducement for 12 h drived rapid accumulation in γH2AX foci number but that failed to descend even after 36 h hydroxyurea inducement (Figures 5a and 5b).

We observed the cell apoptosis by the Annexin-V FITC Apoptosis Detection Kit. As is expected, DFSCs from old donors with 8mM hydroxyurea inducement increased the percentage of apoptosis in DFSCs compared to young donors (Figure 5c and 5d).

### Biological function analysis of the differentially expressed proteins post HU inducement

We found that DFSC with 8mM hydroxyurea not only reduced the capability of proliferation and differentiation but also caused ageing-associated phenotypic changes, suggesting that a premature senescence model was successfully established. However, it was still unclear whether our cellular aging model may evolve bioligical machinery associated with senescence. So, we then continue to confirm the ageing models by observing well-known senescence-related proteins and signaling pathways.

**Figure 6:**
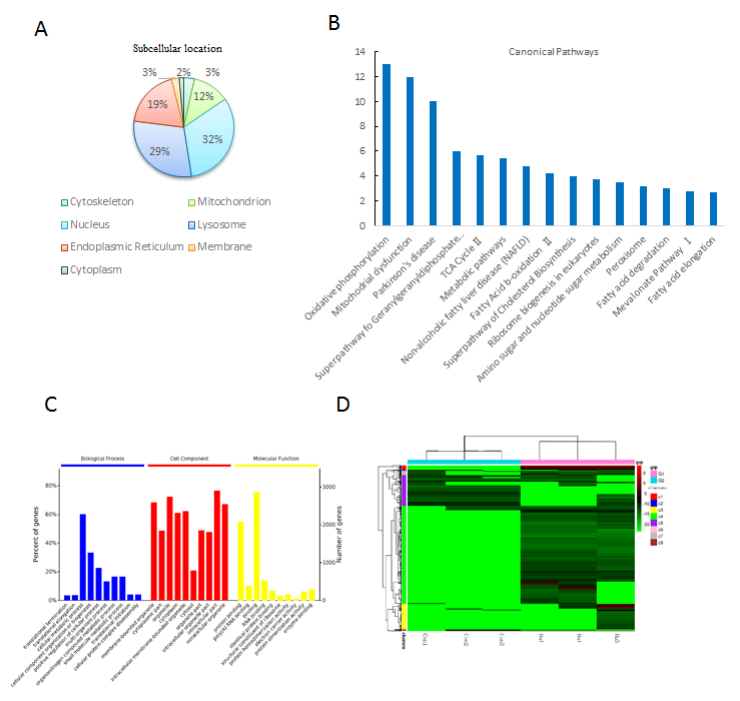
Biological function analysis of the differentially expressed proteins post HU treatment **a**. The cellular component annotations of differentially expressed proteins with GO analysis from DAVID database. **b**. Top canonical pathways altered post HU treatment with mitochondrial dysfunction being the most significant one. **c**. GO annotation of identified age-related proteins in three categories: biological process (BP), cellular component (CC) and molecular function (MF). **d**. Heat map visualization of significantly regulated probe sets. A hierarchical clustering was analyzed on the probe sets and gene transcripts were selected from 6 chips in 2 arrays (Con and HU treatment) through a filter criteria of at least 2-fold changes with *P*≤0.05 (F test). Columns: samples; rows: genes; color key indicates gene expression value, green: lowest, red: highest.

Here we performed a transcriptome-wide characterization of DFSCs with or without hydroxyurea treatment by RNA sequencing. Transcriptome analysis unraveling key molecular pathways associated with DFSC senescence, and we then use biological tool including Ingenuity Pathway Analysis (IPA) (http://www.ingenuity.com) and DAVID Bioinformatics Resource 6.7 (http://david.abcc.ncifcrf.gov/) to present Bioinformatics observation. The major different expressions are located in the nucleus (32%), the lysosome (29%) and the mitochondrion (12%) (Figure 6a). Furthermore, these genes play a key role in the canonical pathways associated with energy metabolism, mitochondrial dysfunction and ageing (Figure 6b). There are five enriched biological processes (*P* < 0.001) including cellular metabolic process, cellular component organization or biogenesis, positive regulation of cellular process, organonitrogen compound metabolic process and small molecule metabolic process (Figure 6c). Cell component majorly included organelle, intracellular part, membrane-bounded organelle, intracellular organelle and cytoplasm. For molecular function, binding, protein binding, RNA binding, poly (A) RNA binding and enzyme binding were among the top (Figure 6c). Differentially expressed genes between Con and hydroxyurea treatment of the human DFSCs were visualized as heatmaps for RNA-seq (Figure 6d).

### Candidate genes and pathways identified through the DFSC cellular aging model associated with aging characteristics

**Figure 7:**
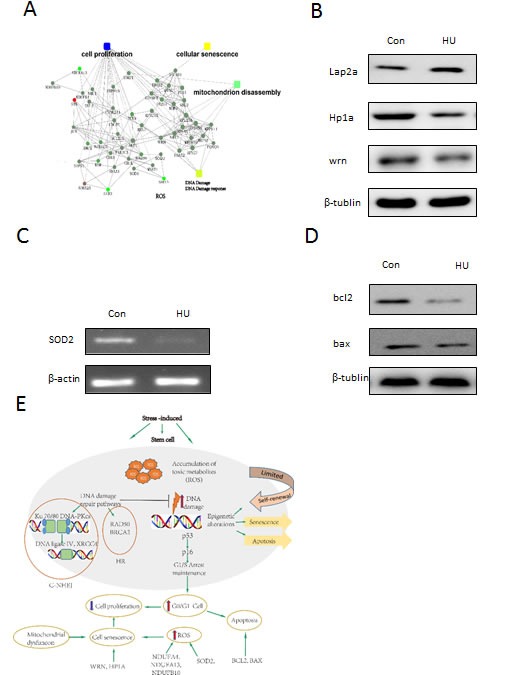
Candidate genes and pathways identified through the DFSC cellular aging model associated with aging characteristics **a**. The key regulatory networks underlying the HU-induced aging model. Proteomic data were imported into the IPA and interacting pathways were constructed in protein expression. **b**. Western blotting analysis of lap2a, hp1a and wrn expression. **c**. Verification of SOD2 expression by RT-PCR with β-actin as an internal control. The data are presented as mean±S.E. from three independent experiments. **d**. Western blotting analysis of bal2 and bax expression. **e**. The schematic diagram outlines the key players and pathways that may mechanistically contribute to the phenotypes in our DFSC aging model.

The establishment of cellular senescence model makes us to utilize genome-wide approach and systematically identify the machinery that recapitulate the associated with decreases in an unbiased manner. To demonstrate such utilities, we presented quantitative transcriptome analysis and found explore main genes and pathways modulating the senescence progression. Functionally, of these genes are majorly related to DNA damage response, repair, cell cycle arrest, cellular senescence, Reactive oxygen species and stress response, mitochondrial integrity, cell apoptosis and cell death (Figure7a). Importantly, DFSCs with hydroxyurea inducement, many of the genes underwent expression changes in a greatly correlated manner and readily formed some canonical pathways related to some senescence markers such as superoxide dismutase 1 (SOD1), superoxide dismutase 2 (SOD2), WRN , NADH dehydrogenase 1b subcomplex 9 (NDUFB9), NADH dehydrogenase 1a subcomplex 4 (NDUFA4), NADH dehydrogenase 1a subcomplex (NDUFA13), PTEN Induced Putative Kinase 1(PINK1), and Bax [44–50] as well as deregulated nutrient sensing and metabolisms (oxidative phosphorylation (OXPHOS), tricarboxylic acid (TCA) cycle, Parkinson's disease and Fatty Acid-β-oxidationII) (Figure 7a).

We presented IPA pathway analysis to demonstrate main cell functional networks altered in HU-induced DFSCs, we found that a large number of proteins are greatly interconnected within phenotypic changes. For instance, WRN and superoxide dismutase 2 was declined in the HU-induced group (Figure 7b and 7c), in accordance with the elevated Reactive oxygen species and DFSC ageing. In addition, we determined mitochondrial aging-related genes such as bax and bcl2 (Figure 7d). Furthermore, we draws the vital actors and signaling pathways that can mechanistically contribute to the phenotypic changes in the cellular aging model (Figure 7e).

## DISCUSSION

In the present study, human DFSCs were treated with 8mM hydroxyurea for 12 h and an increased cell senescence model was successfully established. The percentage of senescence-associated-β-galactosidase positive DFSCs in the hydroxyurea treatment significantly increased than in the control group. Similarly, mesenchymal stromal cells (MSC) with replicative exhaustion, doxorubicin treatment, oxidative stress and X-ray irradiation showed an increase β-galactosidase expression [51]. Consequently, we found that hydroxyurea treatment caused cells to enter a state of irreversible cell cycle arrest accompanied by ageing-like morphology, functional alterations and dysfunction of damaged DNA repair mechanism, which is similar to neural stem cells in mouse [52]. The induction of cellular aging depends on the repair pathways involving the p53 and Rb. Meanwhile, the capacity of proliferation and differentiation of DFSCs was also insulted. In addition, chronic accumulation of DNA damage and a decrease in DNA repair ability were accompanied by accelerated intracellular Reactive oxygen species production and declined cellular apoptosis (Figure 3c). A major production of low radiation exposure in human mesenchymal stromal cells in can trigger the cellular ageing, whereas it has been considered as minimal contribution to apoptosis [53]. These data indicate that premature senescence of cultured human DFSCs with HU-induced treatment may demonstrate major components of the vivo ageing and thus can act as a useful ageing model.

Studies have showed that p53-p21 signaling pathway plays a vital part in cellular response to DNA damage [54] and p16INK4A prevents phosphorylation of Rb though cyclin/cdk complexes. Hypophosphorylated Rb prevents cellular proliferation by inhibitory binding to E2F transcription factors, thus preventing them from triggering genic transcription involved in DNA replication and cellular proliferation [55]. Tumor protein p53-p21 and p16INK4A-Rb signaling pathways are involving in cell cycle arrest and cellular ageing [56]. As we expected, the expression of tumor protein p53, p21 and p16INK4A showed a significant increase with mild hydroxyurea treatment by quantitative real-time polymerase chain reaction (Figure 4a) and Western Blotting (Figure 4h). Studies showed p16INK4A was an increased expression in ageing human and murine organs [57] and an elevated expression of tumor protein P53, p21 and p16INK4A all result in declined proliferative capacity and neurogenesis of neural stem cells (NSCs) [58, 59]. In our context, the establishment of cell-cycle arrest was affected by p16INK4A-Rb axis. For instance, DNA damage initially harms cell-cycle process by tumor protein P53-mediated induction of p21, but if insults persist, this triggers p16Ink4a by P38 MAP Kinase-mediated reactive oxygen species production and mitochondrial dysfunction [60, 61]. We then identified a decline in WRN expression in response to the HU-induced (Figure 7b). Furthermore, WRN-deficient mouse stem cells recapitulated the progressive heterochromatin dysfunction and can serve as a useful marker for physiological aging in human stem cells [40]. Mechanistically Werner Syndrome RecQ Like Helicase (WRN) relates to heterochromatin proteins Suppressor Of Variegation 3-9 Homolog 1(SUV39H1) and HP1 Alpha Homolog (HP1a) and nuclear lamina-heterochromatin anchoring protein LAP2b. Targeted knock-in of catalytically inactive SUV39H1 in wild-type mouse stem cells demonstrates increased cell ageing, resembling WRN-deficient mouse stem cells. Moreover, decline in WRN and heterochromatin hallmarks are observed in mouse stem cells from advancing ageing individuals [40]. These results seem to support that WRN play an important role in conserving heterochromatin stability and highlight heterochromatin dysfunction as a common hallmark of human cellular senescence.

We next analysed the regenerative capacity of human DFSCs from physiologically three young (20-years) and three old (55-years) donors, with Hu-induced treatment. Compared to young donors DFSCs, regeneration efficiency was reduced in old donors DFSCs, but remarkably declined in old donors DFSCs (Figure 5a and 5b). The number of γH2AX foci increased in old donors DFSCs, but did not increase further in old donors DFSCs (Figure 5a and 5b), suggesting that the sharp regenerative decrease during Hu-induced treatment cannot be attributed to a diminished DFSC supply. The counterintuitive demonstration revels an adaptive response: DFSCs adopt a compromised state to prevent from being completely excluded sub-lethal lesions including (8mM hydroxyurea inducement) persistent DNA damage, greatly recapitulating the ageing progression. In fact, similar demonstrations have been observed in different stem cell compartments including Human stem cells and mouse neural stem cells [59, 62]. Taken together, the data imply that DNA damage accrual is increased in stem cells with age, and indicates that proliferative precursor cells are either repaired more readily or are excluded on damaged DNA accumulation.

To test the effect of non-homologous end joining on DFSCs functional capacity with Hu-induced treatment, we demonstrated several key genes of non-homologous end joining pathway by QRT-PCR, including X-Ray Repair Cross Complementing 6 (Ku70), Repair Cross Complementing 4 (xrcc4) and DNA ligase IV (LigIV). As we know, X-Ray Repair Cross Complementing 6 (Ku70) and X-Ray Repair Cross Complementing 5 (Ku80) triggers DNA-PKcs, and together the three proteins form the DNA-PK holoenzyme. DNA-PK activates Artemis, which serves as an endonuclease to progression ends that may not be directly rejoined. Finally, X-Ray Repair Cross Complementing 4(xrcc4) and DNA Ligase IV, which are likely recruited by Ku, function together to catalyze end ligation itself. Non-homologous end joining often occurs concomitant with loss of a few nucleotides at the site of joining [63]. The expression of Non-homologous end joining pathway genes declined with Hu-induced treatment (Figures 4a and 4k), which might be underlying HU-treated persistent DNA damage, resulting in DFSC ageing. In addition, we then observed the importance of hydroxyurea function in stem cell by western blot. Similarly, the expression of HR pathway genes (Brca1 and xxrc2) increased with Hu-induced treatment (Figures 4b), Moreover, proliferate capacity of DFSCs from young donors had reduced with hydroxyurea treatment, which was significantly exacerbated with age (Figure 5a and 5b) and was related to increased apoptosis (Figure 5c and 5d). Cumulatively, these data identify some major genes in maintaining DFSC function by maintaining the capacity of reconstitution potential, self-renewal, proliferation and stem cell viability under stresses.

Elevated production of reactive oxygen species has long been considered another major marker in our DFSC aging model (Figures 3c and 3d). The result show that the level of superoxide dismutase 2, mitochondrial (SOD2) expression is a significant decrease in HU-induced DFSCs (Figures 7c). As we know, SOD2 traditionally scavenges Reactive oxygen species in the inner mitochondrial matrix and serves as a major defense against mitochondrial stress [64]. Velarde’ Studies in the skin suggest that SOD2 deficiency lead to mitochondrial oxidative damage and further triggered cellular senescence and senescence phenotypes [65]. Recent study, WRN-deficient MSCs recapitulated main phenotypes of premature senescence, containing premature lack of proliferative potential, higher number of senescence-associated-β-galactosidase positive cells, upregulated expression of senescence-associated genes p16Ink4a and p21Waf1, and activation of aging-related secretory phenotype (SASP) [40]. In addition, NADH dehydrogenase 1a subcomplex 4 (NDUFA4), NADH dehydrogenase 1a subcomplex13 (NDUFA13) and NADH dehydrogenase 1a subcomplex10 (NDUFB10), components of the mitochondrial complex I, were greatly downregulated in HU-induced DFSCs (Figure 7e) and their deficiency was related to mitochondrial dysfunction [47]. Accumulating mitochondrial damage is key element for an increase level of Reactive oxygen species with age in a self-perpetuating cycle [66]. ROS-induced mitochondrial insult further led to higher Reactive oxygen species production that resulted in even more mitochondrial damage accumulation [20]. As unraveled by our transcriptome analysis, lots of genes deregulated in the cellular senescence model are associated with mitochondrial oxidative stress (Figure 7a), suggesting the importance of mitochondrial stabilization for senescence. Furthermore, our data found that the level of Apoptosis Regulator Bcl-2 and BAX expression is a decline in HU-induced DFSCs, which seem to support that the BCL-2 family genes are the major mediators of the intracellular apoptotic signaling pathways because they regulate the commitment of the cells to mitochondrial outer membrane permeabilization (MOMP) [67].

The accelerated Reactive oxygen species levels might also degrade the DNA damage response/repair [68]. The transcriptome analysis showed that in HU-induced DFSCs the top enriched genes is associated with DNA replication, DNA recombination, DNA repair, Reactive oxygen species, energy production and nucleic acid metabolism (Figure 7a). As we know, Reactive oxygen species are linked to several cellular metabolic and signaling progressions [69]. Our observation with the DFSC ageing model reveal that the Reactive oxygen species signaling pathways might perform in concert with differences in oxidative phosphorylation (OXPHOS), tricarboxylic acid (TCA) cycle, Parkinson's disease and Fatty Acid β-oxidationII (Figure 6b), resulting in deregulated nutrient sensing and metabolism, and consequentially increased ageing.

Mediation of oxidative metabolism has further been involved in stem cell senescence through studies of the sirtuin family of NAD-dependent protein deacetylases, which are key mediators of oxidative stress, ageing and stem cell function. For instance, recent studies show a role for Sirtuin 1 in conserving mesenchymal stem cell proliferation and differentiation, which have been suggested to decrease with age [70, 71]. Also, the expression of BAX was greatly downregulated in HU-treated DFSCs by the proteomic analysis (Figure 7d), which may be activated and translocated to the mitochondria to initiate mitochondrial dysfunction and apoptosis.

In conclusion, to understand the significance of these changes in dental follicle stem cells with aging, we have developed a protocol and established a cellular senescence model using DFSCs that might detailly demonstrate the ageing changes *in vivo*, we have tested serial experiments by this model including some major genes related to persistent DNA damage, cellular ageing, G1/S cell cycle arrest, elevated reactive oxygen species and declined proliferative and differentiative capability, and regenerative process. Our data also showed that DFSCs from young are more resistant to hydroxyurea due to declined apoptosis, increased Non-homologous end joining ability, and more-rapid DNA repair. The data has enabled it possible to explore DFSC ageing machinery detailly. The present demonstration that age-relative phenotype in different age donors, DFSC function in an age-dependent manner under stressful conditions shows that DNA damage accrual can underlie the declined capability of stem cells to mediate a return to homeostasis after exposure to injury or stress. Importantly, the use of genetically determined cellular senescence model of specific molecular causes of ageing and proof-of-principle assays in cellular senescence model will make it present the ageing mechanisms. The studies could ultimately offer a rational basis to predict which approaches of stem cell-directed therapies are most promising to improve organ maintenance and insult tumorigenesis in the associated disorders and ageing humans.

## MATERIALS AND METHODS

### Human dental follicle stem cell culture and identification

Primary DFSCs were derived from donors. The dental follicle cell was isolated and enzymatically dissociated in Hank's balanced saline solution buffer (HBSS) (WISENT, Canada) containing 1 mg/ml trypsin (WISENT) at 37°C for 10 min followed by 5 min of centrifugation at 350g upon trypsin inhibition (Invitrogen). The isolated cells were washed with HBSS and resuspended in DMEM/F12 medium (WISENT). DFSC formed over 7 days of incubation with 5% CO2 at 37°C. Subculturing was done every 3-4 days. Characterisation of DFSCs was performed using Human MSC Analysis Kit (BD) by flow cytometry (Unpublished data were provided by H.W.). Experiments were performed with cultured cells between passages 2 and 12 except those otherwise indicated.

### γH2AX immunostaining

Cells were seeded at 1×10^5^ cells per ml in 24-well plates (Trueline, USA), treated with hydroxyurea (Sigma, St. Louis, MO, USA) at a concentration of 0.5, 8 or 20 mM, and incubated at 37°C until the collection time points.

γH2AX was showed by using the SCIP hos (single-cell imaging of phosphorylation) assay [72]. In a word, DFSC cells were sorted into droplets of PBS on poly (L-lysine)-coated slides, then fixed, permeabilized and stained with phospho-specific (Ser 139) histone H2AX antibody (Abcam). After being washed, the cells were stained with a secondary Alexa Fluor 488-conjugated antibody and 4, 6-diamidino-2-phenylindole. Quantification of γH2AX foci was performed by fluorescence microscopy and analysed statistically with the Mann-Whitney U-test.

### Clonogenic assay

Two replicates of 200 cells were seeded in two six-well culture dishes with hydroxyurea (8mM) and without hydroxyurea medium as that for cell culture and cultivated for 10 days in a humidified atmosphere at 37°C and 5% CO2. Medium was changed every fourth day. Colonies were visually scored using an inverted microscope.

### Osteogenic, adipogenic and chondrogenic differentiation

Osteogenic, adipogenic and chondrogenic differentiation of DFSCs was simultaneously performed under the same differentiation conditions as described before [73, 74]. After three weeks, osteogenic differentiation was analyzed by Alizarin Red staining and quantified with a method based on acetic acid extraction and neutralization with ammonium hydroxide [75]. A Tecan infinite M200 plate reader was employed to measure the absorbance at 405 nm.

At day 14 after induction of adipogenic differentiation, the cells were stained with Oil Red O (Sigma, St. Louis, MO). Cells were washed twice with phosphate-buffered saline (PBS) and fixed with 3.7% formaldehyde in PBS for 30 min and then washed twice with PBS. Cells were stained with 0.35% Oil Red O dye in isopropyl alcohol for 30 min. Excess stain was removed by washing with 70% ethanol and PBS. Visualization of intracellular lipid accumulation was achieved via Oil Red O staining.

Chondrogenic differentiation was induced in micromass culture for three weeks. Subsequently the pellets were fixed with 10% formalin and paraffin embedded. 1 mm sections were stained with Alcian blue in combination with PAS (Periodic acid-Schiff) in an automated slide stainer and photo-documented.

### Senescence-associated-β-galactosidase assay

Cellular senescence was determined by senescence-associated-β-galactosidase staining. Staining was performed using the Senescence Cells Histochemical Staining Kit (Sigma) according to the manufacturer's guidelines. Positive staining was evaluated after 12-16 h incubation at 37°C in a CO_2_-free atmosphere. The blue stained cells from 10 different fields were counted with results presented as a percentage of positive cells.

### Reactive oxygen species measurements

Cells were cultured in six-well plates (Corning Incorporated, Corning, NY) in regular growth media until reaching 70-80% confluence. Then, cells were treated with hydroxyurea (8mM) and without hydroxyurea. After incubation with agents, cells were harvested and incubated for 30 min with dyes to detect Reactive oxygen species [10 μM dihydroethidium (DHE) and 0.5-10μg/ml 4, 6-diamidino 2-phenyl-indole (DAPI)]. Cells were then washed twice with 1% BSA/PBS and analyzed for fluorescence microscopy.

### RNA extraction and quantitative real-time polymerase chain reaction (qRT-PCR)

Total RNA was isolated from the cells at the logarithmic phase by Trizol technology (Tiangen Biotech Co., Ltd., Beijing, China). For the mRNA analysis, the cDNA primed by oligo-dT was made with a prime Script RT reagent kit (Tiangen Biotech Co., Ltd., Beijing, China) and the mRNA level of the genes HOXC9 was quantified by a duplex-qRT-PCR analysis where the Taqman probes in a different fluorescence for the β-actin (provided by Shing Gene, Shanghai, China) were used in the FTC-3000P PCR instrument (Funglyn Biotech Inc, Canada). Using the 2−ΔΔCt method, the normalization with the β-actin level was performed before the relative level of the target genes was compared. The sequences of primers used for the qRT-PCR (Table 1).

**Table 1:** Primers used for qPCR

Primer name	Primer sequence (5’–3’)	Use
P53-F	5’ GTTCCGAGAGCTGAATGAGG 3’	qPCR
P53-R	5 ’ TCTGAGTCAGGCCCTTCTGT 3′	qPCR
P21-F	5' GCCCGCTCTACATCTTCTG3'	qPCR
P21-R	5' GTGCCATCTGTTTACTTCTCAA3'	qPCR
P16-F	5' GACATCCCCGATTGAAAGAA3'	qPCR
P16-R	5' CCTGTAGGACCTTCGGTGAC3'	qPCR
BRCA1-F	5' CTTCTACCAGGCATAT 3'	qPCR
BRCA1-R	5' TTGGCTTGTTACTCTT 3'	qPCR
XXRC2-F	5' TGACTATCGCCTGGTT 3'	qPCR
XXRC2-R	5' GGTTGCTGCTTTGAGA 3'	qPCR
KU70-F	5' AGGAGTCGCTGGTGATTG 3'	qPCR
KU70-R	5' CACCTGCTCTGGAGTTGC 3'	qPCR
XXRC4-F	5' GCAGAATCCACCTTGT 3'	qPCR
XXRC4-R	5' CCTGCTCCTGACAACA 3'	qPCR
LIGASE4-R	5' TGGTGCTTCTCCTACT 3'	qPCR
LIGASE4-F	5' CAAACTTAGTTCCCTTT 3'	qPCR
SOD2-F	5' GCGGGCGTTTACTCTTAG 3'	qPCR
SOD2-R	5' TCTCCTCGGTGACGTTCA 3'	qPCR
β-actin- F	5’ TTTTCCAGCCTTCCTT 3’	qPCR
β-actin-R	5’ TTGGCATACAGGTCTTT3’	qPCR

β-actin was used as housekeeping genes

### Cell cycle analysis

DFSCs cells were harvested from the culture flasks and centrifuged at 1,000 rpm for 5 min. The pellet was resuspended in fresh media with hydroxyurea (8mM) and a control with only media. DFSCs cells were then cultured for 24h in an incubator at 37 °C with 5% CO2. At the end of the culture, DFSCs cells were centrifuged at 1,000 rpm for 5 min and the pellet was resuspended in 200 ul phosphate buffered saline (PBS). The cells were fixed by adding 400 ul of 200 proof ethanol (final percentage at 66%, Sigma Aldrich) and incubated for 15 min on ice. The cells were then centrifuged at 1,500 rpm for 5 min and the pellet was resuspended in 200 ul propidium iodide (PI) solution containing 50 ug/ml PI (Biotium, Hayward, CA), 0.1 mg/ml RNase A (Sigma-Aldrich), and 0.05% Triton X-100 (Sigma-Aldrich). DFSCs cells were incubated for 40 min at 37°C before performing flow cytometry analysis.

### Cell apoptosis analysis

Cells were harvested and rinsed with PBS twice. Then 5 μl of FITC-labeled enhanced annexinV and 5 μl (20 μg/ml) of propidium iodide were added into 100 μl cell suspension. Upon incubation in the dark for 15 min at room temperature, samples were diluted with 400 μl PBS. Apoptosis was determined by the Annexin-V FITC Apoptosis Detection Kit, which was performed according to the manufacturer's instruction (BD Pharmingen). Data were acquired on a FACS Calibur flow cytometer (BD Biosciences). Results were obtained by analyzing data with FlowJo Version 7.6.1 software (TreeStar). Results represent the mean value of three independent experiments. The result was analyzed according to the manufacturer's instruction. The experiments were performed independently three times and a representative was shown.

### Western blot analysis

Cultured DPSCs were washed with ice-cold PBS and lysed with 2 X SDS lysis buffer. Protein concentrations were determined by the BCA protein assay kit (Pierce, Rockford, IL, USA). Proteins were separated on 10% SDS-polyacrylamide gels (Bio-Rad Laboratories, Inc., Hercules, CA, USA) and transferred to a PVDF membrane (Millipore, Billerica, MA, USA). Membranes were blocked in 5% non-fat milk powder in TBS-T (0.1% Tween-20 in PBS), and incubated with primary antibodies overnight at 4°C. After washing in PBS-T, membranes were incubated with HRP-conjugated secondary antibodies (Invitrogen) for 1 h at room temperature with signals detected using ECL Super Signal (Pierce). Quantifications were done using Image-Quant Software (Molecular Dynamics, Sunnyvale, CA, USA). The primary antibodies used include rabbit anti-p16INK4A (Abclonal Technology, Wuhan, China), anti-p21 (Abclonal Technology, Wuhan, China), anti-p53 (Abcam), anti-ku70 (Abcam), anti-Xrcc2 (Abclonal Technology, Wuhan, China), anti-ligase4 (Abclonal Technology), anti-brca1 (Abclonal Technology),anti-Xrcc4 (Abclonal Technology) and mouse anti-β-tublin (Abclonal Technology, Wuhan, China), WRN (Abcam), Hp1a (Abcam), Lap2a (Abcam), BCL2 (Abcam), BAX (Abcam).

### RNA sequence analysis

### RNA sequencing library construction and Illumina sequencing

Isolation and enrichment of mRNA from total RNA was performed using oligo (dT) magnetic beads (Illumina, CA, USA). Then, mRNA was fragmented to short fragments to be used as templates for randomhexamer-primed synthesis of first strand cDNA by fragmentation buffer. Second-strand cDNA was synthesized using buffer, dNTPs, RNase H, and DNA polymerase I. A paired-end cDNA library was synthesized using the Genomic Sample Preparation Kit (Illumina,CA, USA) according to themanufacturer's instructions. Short fragments were purified with QIAQuick1 PCR extraction kit (QIAGEN, Germany) and eluted in 10 μL of EB buffer (QIAGEN, Germany). These short fragments were connected via sequencing adapters (Illumina, CA, USA). Agarose gel electrophoresis was used to select fragments approximately 50 bp in size. Finally, cDNA libraries were sequenced on an Illumina HiSeq™ 2500 (Novogene, Beijing).

### Protein pathway analysis

We annotated our database with G.O. terms for cellular components, cellular processes, and cellular functions. GO annotation of the identified proteins was done using DAVID (V6.7:http://david.abcc.ncifcrf.gov/). Differentially expressed proteins were analyzed using IPA (Ingenuity Systems:http://www.ingenuity.com). In addition, the overrepresented biological functions, molecular networks and canonical pathways were generated based on information in the Ingenuity Pathways Knowledge Base.

### Statistical analysis

Data were analyzed by Student's t-test and one-way analysis of variance (ANOVA) followed by post hoc multiple comparison tests. Significance was accepted at P < 0.05.
